# Association between serum albumin and mortality in Japan older people with dysphagia

**DOI:** 10.1038/s41598-022-16010-y

**Published:** 2022-07-15

**Authors:** Gaigai Li, Xun Zhou, Xunrui Hou, Yuheng Luo, Danmao Li, Tongtao Fan

**Affiliations:** 1 Department of Respiratory and Critical Care Medicine, The Second Affiliated Hospital of Guizhou University of Traditional Chinese Medicine, Guiyang, China; 2 Department of Acupuncture and Massage, The Affiliated Hospital of Guizhou Medical University, Guiyang, China

**Keywords:** Diseases, Oral diseases

## Abstract

To determine whether there is a link between serum albumin and mortality among participants in the elderly in Japan. This is a single-center,retrospective cohort study analysis of 253 old patients with dysphagia from Japan, conducted from January 2014 to January 2017. The primary outcome was mortality. We performed Cox regression analysis to compare the mortality between the two groups (divided by serum albumin = 3 g/dl). 253 patients were included in the analysis, of whom the number of serum albumin under 3 g/dl was 93. The log-rank test showed a significant longer mortality in the high group (serum albumin >  = 3 g/dl) compared with the low group (median, 382 vs. 176 days, *P* < 0.0001). Cox regression analysis showed that unadjusted HR for the high group relative to the low group was 0.40 (95% CI: 0.29–0.57; *P* < 0.001). After adjusting 3 models in multivariable analysis, serum albumin was significantly associated with mortality. The adjusted HRs (95% CI) for total mortality rates were 0.46 (0.33–0.65), 0.66 (0.44–0.99) and 0.64 (0.42–0.97), from model 2 to model 4. There is negative association between serum albumin and mortality in Japanese old people with dysphagia.

## Introduction

Dysphagia, or disordered oropharyngeal swallowing, is a common finding in elderly persons^1^ whether are institutionalized or living in the community^2,3^, which is caused by age-related physiological changes in swallowing, including a decline of swallowing function and decreased digestive tract motility due to decreases in muscle mass and connective tissue elasticity^4,5^. Cicheroet^6^ reported a 25–30% prevalence among acute inpatients who underwent clinical screening for dysphagia. Dysphagia may lead to malnutrition and dehydration ^5^. It is a risky factor for pneumonia^7^ and stroke ^8^.

Serum albumin is a widely used routine clinical test and serves as a biomarker of inflammatory and nutritional status^9–11^, which is a reliable index of malnutrition. The effect of inflammation on albumin levels is responsible for much of the morbidity and mortality associated with hypoalbuminemia^9^. Some of the prior studies reported serum albumin was associated with chronic kidney disease and cardiovascular^12–14^, which is also a risky factor for dysphagia in elderly hip fracture surgery patients^15^. Hypoalbuminemia is strongly associated with mortality^11^, and is an independent determinant of poor outcome following acute ischemic stroke^16^.

Although some studies have shown that serum albumin is associated with many diseases, but few studies have revealed the association between serum albumin and mortality in elderly swallowing patients. A previous propensity-matched cohort study shows PEG (percutaneous endoscopic gastrostomy) is associated with a significantly longer survival time in older persons with dysphagia^17^. We perform a secondary analysis of the cohort study data to evaluate whether there is a link between serum albumin and mortality among participants in Japan.

## Patients and methods

### Study population and design

This study was a single-center, retrospective cohort study. Consecutive older patients with dysphagia who received PEG or TPN (total parenteral nutrition)^18^ between January 2014 and January 2017 in Japan. People who had advanced cancer or required a PEG for gastric decompression were excluded. Patients who had a PEG inserted before January 2014 were also excluded.

Because of the anonymous nature of the data, the requirement for informed consent was waived. All methods were performed in accordance with the relevant guidelines and regulations. The present study was approved by the Ethical Review Board of Miyanomori Memorial Hospital and was exempted from informed consent requirements owing to its retrospective design.

### Procedures

The decision to select PEG feeding or TPN was made after sufficient discussion, including patients or their family and clinicians. Appropriate nutrition was administered based on clinical evaluation by clinicians. Clinical details were obtained from patients’ medical records including age, gender, height, weight, underlying diseases, and blood test results.

### Laboratory assays

The blood test results were performed within 7 days before the start of PEG feeding or TPN. Body mass index (BMI) was calculated using the height and weight measured on admission. Daily calorie was investigated on the seventh day after the procedure in both groups.

### Outcomes

The primary outcome was defined as mortality after the start of the procedure during the follow-up period.

### Statistical analysis

All normally distributed and skewed continuous variables were described as mean (SD) or median (interquartile range [IQR]). Categorical variables were expressed as frequencies (%). Baseline characteristics are presented according to the serum albumin by 3 g/dl. Multivariable Cox regression analysis were adopted to assess the independent association between serum albumin and mortality. An extended Cox model approach was used for different covariates adjusted models. Survival curves were plotted by Kaplan–Meier analyses. These potential confounders were chosen on the basis of previous scientific literature, or a more than 10% change in effect estimates. All the analysis were performed with the statistical software packages R (http://www.R-project.org, The R Foundation) and Free Statistics software versions 1.2.

## Results

### Study participants and baseline characteristics

A total of 253 patients were included in the analysis, of whom the number of serum albumin below 3 g/dl was 93.Among them, there are 99 males and 154 females. 180 of whom underwent PEG feeding and 73 of whom underwent TPN. The median length of follow-up for censored cases was 601 days (range, 404–823 days). The mean age was 84.8 years old (SD 7.1) in low serum albumin group. The mean hemoglobin value was 9.8 g/dl (SD 1.9) in low serum albumin group. The median of survival time was 306 days. Baseline clinical and biochemical characteristics of participants were stratified by serum albumin (3 g/dl) in Table 1.

**Table 1:** Baseline characteristics of patients.

Variables	Alb < 3 g/dl (n = 93)	Alb ≥ 3 g/dl (n = 160)	*p*
PEG	52 (55.9)	128 (80)	< 0.001
Age(yr)	84.8 ± 7.1	82.1 ± 10.3	0.027
sex(male)	42 (45.2%)	57 (35.6%)	0.172
Cerebrovascular diseases	40 (43%)	93 (58.1%)	0.028
Severe dementia	50 (53.8%)	52 (32.5%)	0.001
Neuromuscular diseases	2 (2.2%)	12 (7.5%)	0.131
Aspiration pneumonia	46 (49.5%)	48 (30%)	0.003
Ischemic heart diseases	22 (23.7%)	25 (15.6%)	0.157
Chronic heart failure	48 (51.6%)	59 (36.9%)	0.031
Chronic lung diseases	10 (10.8%)	9 (5.6%)	0.213
Chronic liver diseases	8 (8.6%)	7 (4.4%)	0.273
Chronic kidney diseases	31 (33.3%)	22 (13.8%)	< 0.001
Total lymphocyte count (mm^3^)	137.1 ± 39.5	167.2 ± 36.2	< 0.001
Hemoglobin (g/dl)	9.8 ± 1.9	11.7 ± 1.8	< 0.001
Total cholesterol (mg/dl)	970	1329	< 0.001
	(735.8–1322.0)	(999.4–1663.0)	
C-reactive protein (mg/dl)	2.6	0.5	< 0.001
	(1.4–6.1)	(0.2–1.4)	

Values of total cholesterol and C-reactive protein are median (IQR). Values of age, total lymphocyte, and hemoglobin are given in Mean ± Std. Values of other variables are given in numbers (%).

*PEG* percutaneous endoscopic gastrostomy.

### Kaplan–Meier curve

**Figure 1:**
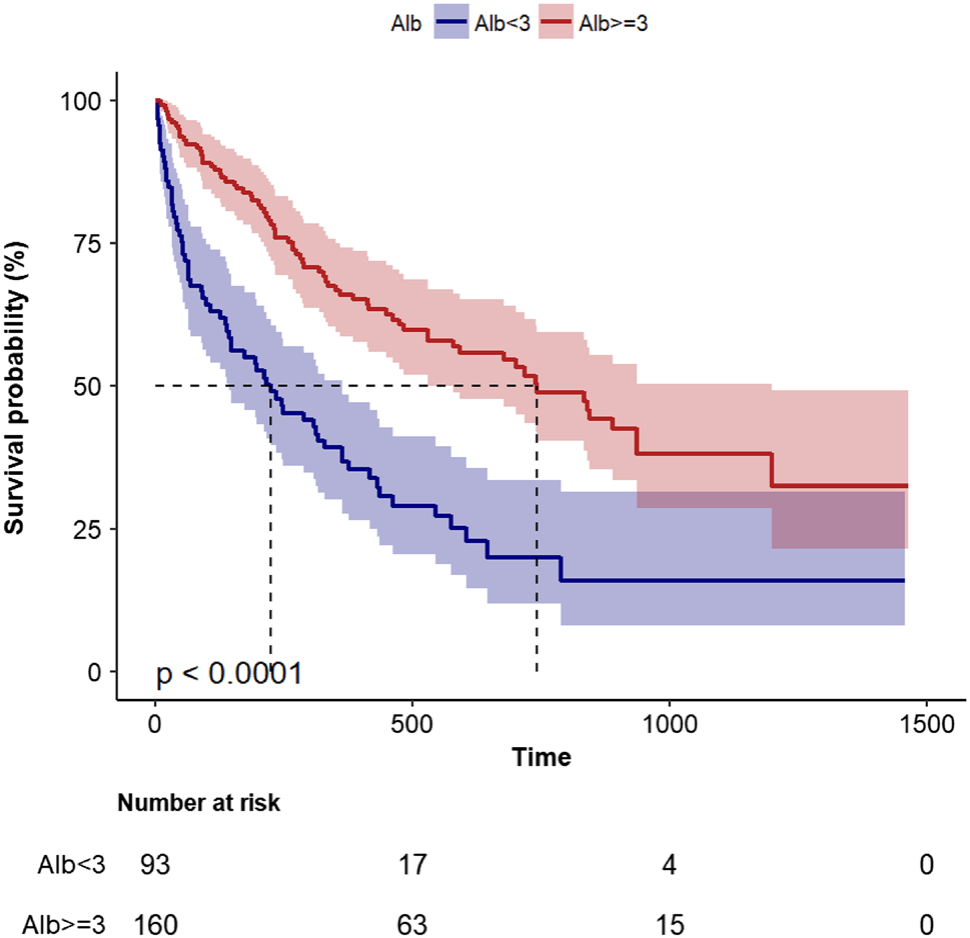
Kaplan Meier curve of time to death during follow-up.

The Kaplan–Meier curve is illustrated in Fig. 1. The log-rank test shows a significantly longer mortality in the high group (serum albumin >  = 3 g/dl) compared with the low group (median, 382 vs. 176 days, *P* < 0.0001).

### Association between serum albumin and mortality in different models

Table 2 shows that unadjusted HR for the high group relative to the low group was 0.40 (95% CI: 0.29–0.57; *P* < 0.001). After adjustment in multivariable analysis in Table 2, serum albumin was significantly associated with mortality, so that survival time was longer in Alb ≥ 3 compared with Alb < 3 group. Furthermore, additional adjustment for age, sex, PEG, cerebrovascular diseases, severe dementia, neuromuscular diseases, aspiration pneumonia, chronic heart failure, total lymphocyte count (mm^3^), and c-reactive protein (mg/dl) did not substantially change the results. The adjusted HRs (95% CI) for total mortality rates were 0.46 (0.33–0.65), 0.66 (0.44–0.99) and 0.64 (0.42–0.97), from model 2 to model 4.

**Table 2:** Association between serum albumin and mortality in different models.

Variable	Model 1		Model 2		Model 3		Model 4	
	HR	*P*	HR	*P*	HR	*P*	HR	*P*
Alb	0.38 (0.29–0.51)	< 0.001	0.44 (0.33–0.6)	< 0.001	0.61 (0.42–0.9)	0.012	0.61 (0.41 ~ 0.91)	0.015
Alb < 3	1(Ref)		1(Ref)		1(Ref)		1(Ref)	
Alb ≥ 3	0.4 (0.29–0.57)	< 0.001	0.46 (0.33–0.65)	< 0.001	0.66 (0.44–0.99)	0.043	0.64 (0.42–0.97)	0.034

Model 1 is not adjusted. Model 2 adjusts for age and sex. Model 3 adjusts for model 2 plus PEG, CHF, TC, dement, asp, and CRP. Model 4 adjusts for model 3 plus CI, NMD.

## Discussion

In this study, we found that lower levels of serum albumin (< 3 g/dl) were associated with an increased risk of mortality. The association persisted after adjusting for different models. The elderly people are more likely to suffer malnutrition due to comprehensive factors, from physiological changes, such as appetite loss, changes in taste, depressive symptoms, to social and economic factors such as income, living circumstances and lifestyle^19–21^. Malnutrition in elderly was associated with higher mortality risk^22^ and poor Quality of life^23^.

Serum albumin, a protein which is synthesized in the liver^24^, is a marker of nutritional status of older people^25,26^. Serum albumin has antioxidative properties^27^ and low serum concentrations that have been suggested to be an indicator of inflammation, hypercoagulable states and liver disease^28,29^. The levels of serum albumin are associated with various diseases, although some studies do not provide direct evidence^30^, other studies report that a lower concentration of serum albumin is associated with cardiovascular mortality, acute respiratory distress syndrome , acute stroke, chronic kidney disease and other complications^12–14,31,32^. Indeed, a meta-analysis showed that hypoalbuminemia was an independent predictor of poor outcome^33^, which appeared to be independent of both nutritional status and inflammation. A study showed that low admission serum albumin was a prognostic determinant of 30 day case fatality and adverse functional outcome following acute ischemic stroke^34^. Meanwhile, high serum albumin has a protective effect on healthy older persons who do not have evidence of cytokine-mediated inflammation^32^.

On one hand, serum albumin may reflect the nutritional state of the human body. On the other hand, we can use proserum to supply the serum albumin when below 3 g/dl. Protein powders and a high protein diet was needed when the serum albumin was 3–4 g/dl. Albumin administration may improve organ function and in hypoalbuminemic critically ill patients^35^. However, results of the SOAP study^16^ showed ICU and hospital mortality rates were higher in patients who received albumin than those do not. Other two studies revealed the similar results, albumin using showed no benefit in outcome^36,37^. Further studies are needed to clarify the role of albumin in ICU patients.

The subgroup of PEG shows no differences in two groups (Supplementary Material Table 1). Previous studies have shown that enteral nutrition was associated with lower mortality rates^38,39^. Also there is no significant superiority of TPN feeding compared with PEG^40,41^. The FOOD study didn’t support a policy of early initiation of PEG feeding in dysphagic stroke patients^42^. There is strong evidence for not using enteral nutrition (EN) in the first week in dysphagic, and not using volitional nutrition support (VNS) in non-dysphagic stroke patients^43^.

There are some limitations to our study. First, subjects in this study were older people with dysphagia, thus limiting the generalizability of our findings. Some studies reported an inverse association between serum albumin and the incident CKD or CVD in middle-aged adults^12,44,45^. Because all the people in our study were over 50 years old, we could not predict the association of HSA and mortality in the middle aged people. Second, there is a possibility of a selection bias because we only had one measurement of serum albumin within 7 days of hospitalization, without follow-up measurements. We were unable to assess the effect of serum albumin levels at different time periods on outcomes. Third, this was a single-center study, and the size of sample is small, so a larger multicenter sample is needed to confirm this result. Fourth, this was a retrospective observational study, therefore, assignment to each group may have been biased. Information bias and unmeasured confounding could have influenced our results. Fifth, this study was conducted in elderly patients in Japan, it is uncertain whether it will be applicable in other countries, so more research evidences are needed.

## Conclusions

Our results suggest that there is negative association between serum albumin and mortality in older people with dysphagia, implying we should pay more attention to the serum albumin levels of elderly patients, especially for hospitalized patients, monitor them regularly, and correct hypoproteinemia in time.

## Supplementary Information

Supplementary Information.

## Data Availability

The data are available at http://www.Datadryad.org/. which allows researchers to freely download the original data.
