# Supplementary material for: Association between serum albumin and mortality in Japan older people with dysphagia
Source: Sci Rep. 2022 Jul 15;12:12089. doi: 10.1038/s41598-022-16010-y (PMC9287317; doi:10.1038/s41598-022-16010-y)
Supplement: Supplementary file 1 — Supplementary Information. [file 41598_2022_16010_MOESM1_ESM.docx]

|  | | | |  | | |
| --- | --- | --- | --- | --- | --- | --- |
| Suppplementary Table 1 Subgroup analysis between serum albumin and mortality | | | | | |  |
| Subgroup | N | Status（N%） | Adjusted HR（95%CI） | | P.for.interaction |  |
| PEG |  |  |  | | 0.363 |  |
| TPN | 73 | 62 (84.9) | 0.64 (0.36~1.15) | |  |  |
| PEG | 180 | 76 (42.2) | 0.86 (0.46~1.6) | |  |  |
| Sex |  |  |  | | 0.402 |  |
| Male | 99 | 70 (70.7) | 0.8 (0.39~1.65) | |  |  |
| Female | 154 | 68 (44.2) | 0.65 (0.35~1.2) | |  |  |
| Age |  |  |  | | 0.715 |  |
| <85 | 125 | 55 (44) | 1.38 (0.62~3.1) | |  |  |
| ≥85 | 128 | 83 (64.8) | 0.57 (0.35~0.92) | |  |  |
